# Germline genetics of cancer of unknown primary (CUP) and its specific subtypes

**DOI:** 10.18632/oncotarget.7903

**Published:** 2016-03-03

**Authors:** Kari Hemminki, Bowang Chen, Abhishek Kumar, Olle Melander, Jonas Manjer, Göran Hallmans, Ulrika Pettersson-Kymmer, Claes Ohlsson, Gunnar Folprecht, Harald Löffler, Alwin Krämer, Asta Försti

**Affiliations:** ^1^ Division of Molecular Genetic Epidemiology, German Cancer Research Center (DKFZ), Heidelberg, Germany; ^2^ Center for Primary Health Care Research, Lund University, Malmö, Sweden; ^3^ Department of Clinical Sciences, Clinical Research Center, Lund University, Malmö, Sweden; ^4^ Department of Plastic and Reconstructive Surgery, Skane University Hospital, Malmö, Sweden; ^5^ Department of Medical Biosciences/Pathology, University of Umea, Umea, Sweden; ^6^ Clinical Pharmacology, Department of Pharmacology and Clinical Neuroscience, Umea University, Umea, Sweden; ^7^ Centre for Bone and Arthritis Research, Department of Internal Medicine and Clinical Nutrition, Institute of Medicine, Sahlgrenska Academy, University of Gothenburg, Gothenburg, Sweden; ^8^ Medical Department I, University Hospital Carl Gustav Carus, University Cancer Center, Dresden, Germany; ^9^ Clinical Cooperation Unit Molecular Hematology/Oncology, German Cancer Research Center (DKFZ) and Department of Medicine V, University of Heidelberg, Heidelberg, Germany

**Keywords:** hidden primary cancer, SNP, genotype, germline genetics, genetic risk factors

## Abstract

Cancer of unknown primary site (CUP) is a fatal cancer diagnosed through metastases at various organs. Little is known about germline genetics of CUP which appears worth of a search in view of reported familial associations in CUP. In the present study, samples from CUP patients were identified from 2 Swedish biobanks and a German clinical trial, totaling 578 CUP patients and 7628 regionally matched controls. Diagnostic data specified the organ where metastases were diagnosed. We carried out a genome-wide association study on CUP cases and controls. In the whole sample set, 6 loci reached an allelic p-value in the range of 10^−7^ and were supported by data from the three centers. Three associations were located next to non-coding RNA genes. rs2660852 flanked 5′UTR of LTA4H (leukotriene A4 hydrolase), rs477145 was intronic to TIAM1 (T-cell lymphoma invasion and metastases) and rs2835931 was intronic to KCNJ6 (potassium channel, inwardly rectifying subfamily J, member 6). In analysis of subgroups of CUP patients (smokers, non-smokers and CUP with liver metastases) genome-wide significant associations were noted. For patients with liver metastases associations on chromosome 6 and 11, the latter including a cluster of genes DHCR7 and NADSYN1, encoding key enzymes in cholesterol and NAD synthesis, and KRTAP5-7, encoding a keratin associated protein. This first GWAS on CUP provide preliminary evidence that germline genes relating to inflammation (LTA4H), metastatic promotion (TIAM1) in association with lipid metabolic disturbance (chromosome 11 cluster) may contribute to the risk of CUP.

## INTRODUCTION

Cancer of unknown primary site (CUP) can be referred to as ‘an orphan disease’ because it is diagnosed through metastases in various organs and the primary tumor cannot be found in spite of a defined diagnostic work-up [1, 2]. In collected autopsy data, the primary tumor was found in 73% of the cases, most frequently in the lungs (27%), pancreas (24%), liver/bile (8%), and kidney/adrenals (8%) [3]. In the Nordic countries, CUP incidence increased until about 1995-2000, followed by a sharp decline which seems to be continuing [4, 5]. In the USA, the decline in incidence started already in around 1980 [6]. The decline in CUP incidence, which has been opposite to the incidence of most cancers, has implied that the proportion of CUP cases of all cancer has dropped from about 4 to 2%; contributing factors to the decline may include new and better diagnostic methods [4-6]. In Sweden, CUP ranked as the eight most common cancer in men and women in 2012 with an incidence somewhat higher than that of pancreatic cancer [7]. Because of high fatality, CUP ranks the third to fifth among cancer deaths [6, 8, 9]; in Sweden the ranking was fifth in 2010 after lung, colorectal, prostate and breast cancers. In Sweden, the decrease in incidence has been noted for most metastatic locations but particularly for the liver [10]. Among the few known risk factors, heavy smoking conveys a risk of 3.7 and any level of smoking increased the risk for CUP with respiratory system metastases to 4.9 [11, 12]. Alcohol consumption, body mass index, waist circumference, diabetes and low educational level or socio-economic status may be other risk factors [11, 12]. Familial risk is another established risk factor with possible implications for primary sites [13, 14]. CUP was associated with many cancers in family members, including cancers of the lung, liver and kidney.

CUP can be called ‘orphan disease’ also because mechanistic research into CUP causation has been a neglected area. A review summarizing results on chromosomal aberrations, and oncogene and tumor suppressor gene mutations in CUP concluded that these appeared not to differ from those in metastatic primary cancers [15]. Recent mutational screening and genome-wide sequencing efforts have revealed frequent alterations in the receptor tyrosine kinase/Ras signaling pathways allowing possibilities for targeted therapies [16-18]. Hardly any data are available of germline variants that might predispose to CUP. In view of the familial clustering of CUP with other primary cancers, pointed out above [13], it could be speculated that some of the susceptibility genes for these primary cancers may also predispose to CUP (searchable in the GWAS Catalog, http://www.ebi.ac.uk/gwas/home).

In the present study we carried out a genome-wide association study (GWAS) on CUP using patient blood samples identified from Swedish biobanks from two centers and from German CUP clinics.

## RESULTS

**Figure 1:**
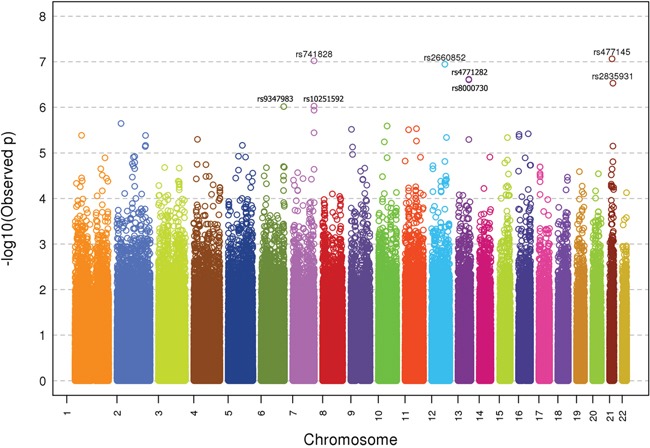
–Log10 p-values for association analysis of DNA from 515 CUP patients and 6227 healthy controls The rs numbers are shown with p-values <10^−6^.

GWAS was successfully conducted on 515 CUP patients and 7226 healthy controls which passed all applied quality control criteria. A Manhattan plot of genotyped SNPs in CUP cases against controls is shown in Figure 1. Eight SNPs in six loci reached a significance level of p<10^−6^. The SNPs, including two linked SNPs on each of chromosomes 7 and 13, are listed by rs numbers in Figure 1.

ORs and p-values of the six unlinked top SNPs (rs9347983, rs741828, rs2660852, rs4771282, rs477145 and rs2835931) are shown in Table 1. While rs9347983, rs741828, and rs4771282 were located either 3′ or 5′ to non-coding RNA genes, rs2660852 was located 5′ to LTA4H (leukotriene A4 hydrolase) on chromosome 12, rs477145 was intronic to TIAM 1 (T-cell lymphoma invasion and metastases) and rs2835931 was intronic to KCNJ6 (potassium channel, inwardly rectifying subfamily J, member 6), both on chromosome 21. Tests for heterogeneity of data from the three centers (p-value for Q > 0.05 and I^2^ < 75.0) showed no heterogeneity for the data on these six SNPs.

**Table 1:** Association data for the most significant SNPs in the case-control study of all CUP patients shown in Figure 1

SNP	Chr	Position	Risk allele*	MAFctrl	OR_het	OR_hom	P_Geno	OR_Allele	P_Allele	P**	I^2^**	Gene	Location	Distance
rs9347983	6	165386880	G	0.24	1.45	1.85	1.1×10^−5^	1.40	1.4×10^−6^	0.46	0.0	RP11-300M24.1	flanking 3′UTR	224kb
rs741828	7	155616377	T	0.25	1.46	2.06	7.2×10^−7^	1.44	1.1×10^−7^	0.19	40.0	Y RNA	flanking 3′UTR	55Kb
rs2660852	12	94969679	C	0.36	1.33	2.50	7.4×10^−7^	1.44	1.9×10^−7^	0.81	0.0	LTA4H	flanking 5′UTR	8.2kb
rs4771282	13	97144122	T	0.39	1.56	1.94	7.4×10^−7^	1.40	1.4×10^−7^	0.35	3.9	RP11-12OE13.1	flanking 5′UTR	16Kb
rs477145	21	31684281	T	0.31	1.47	1.93	1.2×10^−6^	1.42	1.1×10^−7^	0.05	66.9	TIAM1	intron	−50673
rs2835931	21	38043518	T	0.24	1.53	1.89	1.8×10^−6^	1.43	4.0×10^−7^	0.51	0.0	KCNJ6	intron	−34214

* The risk is calculated to the risk allele

** Heterogeneity for data from the 3 centers

**Figure 2:**
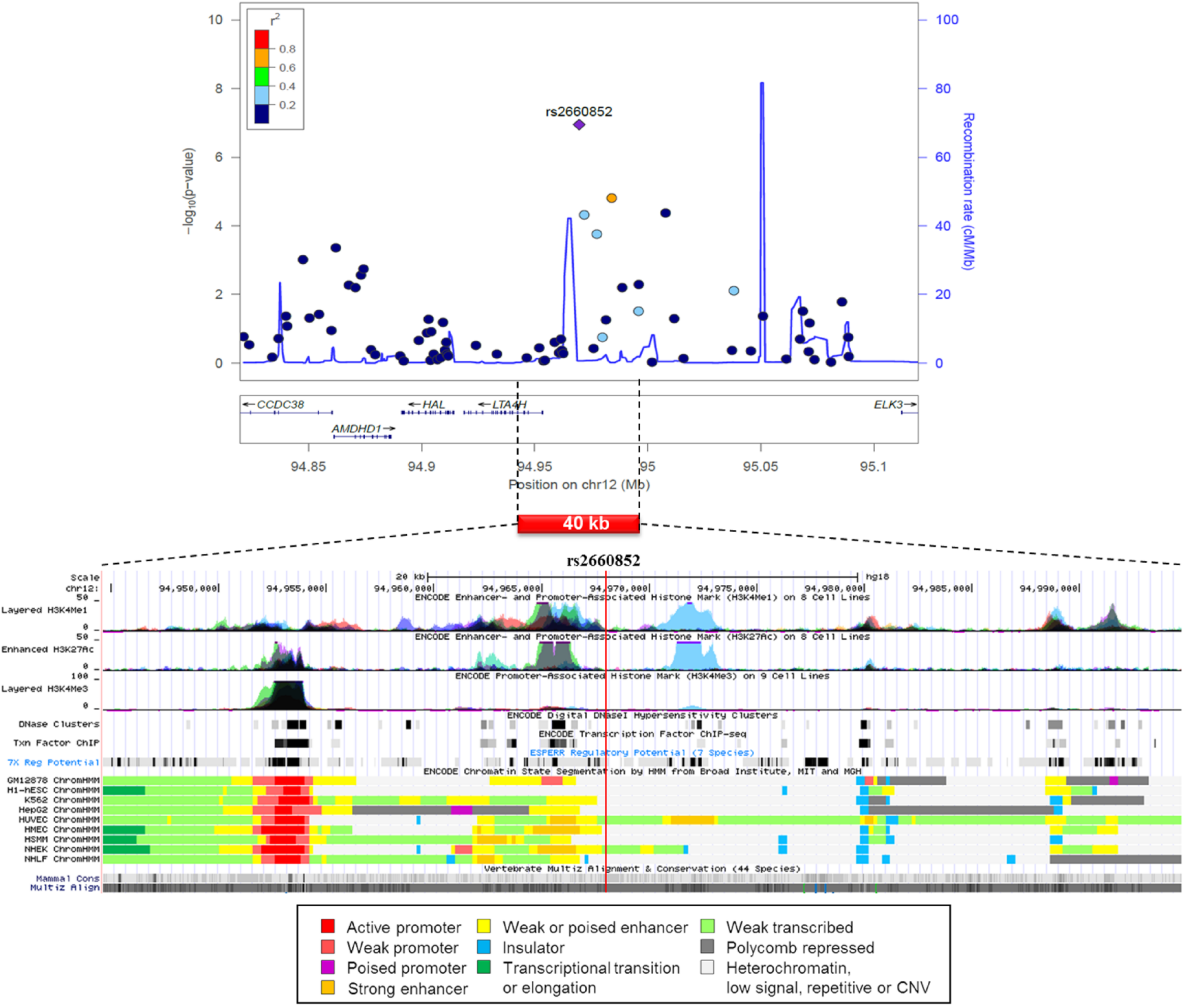
Regional association plot of SNP rs2660852 from 515 CUP patients and 6227 healthy controls with functional annotation based on the ENCODE data

A regional association plot of rs2660852 on chromosome 12 is shown in Figure 2 from 515 CUP patients and 6227 healthy controls together with functional annotation based on the ENCODE data. The SNP is flanking 5′UTR of LTA4H next to a weak or poised enhancer and histone enhancer and promoter marks, constituting the regulatory elements of LTA4H.

Subgroup analyses were carried out by the smoking status, histology and CUP location. Adenocarcinoma was the most common histological type and with 376 cases the data looked essentially as those for all CUP as shown in Figure 1 (data not show). Similarly, the most common location was unspecified (i.e., location unspecified) CUP which had 223 cases and with essential peaks as in Figure 1 (data not shown). However, data for smokers and non-smokers showed some novel associations reaching even genome-wide p-values (p<5×10^−8^) as shown in Table 2. For smokers (258 cases), rs10974489 reached a genotypic p-value of 3.3×10^−12^. The SNP was located 3.8 kb 5′ of the GLIS3 gene (GLIS Family Zinc Finger 3). For non-smokers (210 cases), rs17053186 reached a genotypic p-value of 1.6×10^−9^; this SNP was intronic to CACNA1D (calcium channel, voltage-dependent, L type, alpha 1D subunit). The p-value for rs1514846 was almost equally low 3.4×10^−9^; the SNP was next to an RNA coding gene. CUP with liver metastases (69 patients) showed a highly significant association for SNP rs910609 on chromosome 6 (allelic p-value 5.4×10^−8^) flanking 5′UTR of C6orf223. On chromosome 11 there were linked SNPs rs1790349 with genotypic p-value of 6.2×10^−11^, rs3829251 (9.5×10^−11^), rs10898193 (1.1×10^−10^) and rs11234042 (1.4×10^−10^). The adjacent genes were DHCR7 (7-dehydrocholesterol reductase), NADSYN1 (NAD synthetase 1) and KRTAP5-7 (a gene coding for a keratin associated protein). With the exception of the chromosome 9 SNP in smokers, data from the three centers were homogeneous.

**Table 2:** Association data for the most significant SNPs in the analysis of subgroups of CUP patients

SNP	Chr	Position	Risk allele*	MAFctrl	OR_het	OR_hom	P_Geno	OR_Allele	P_Allele	P**	I^2^**	Gene	Location	Distance
**Smoker**														
rs10974489	9	4342222	C	0.28	4.35	2.13	3.3×10^−12^	2.63	1.0×10^−9^	0.00	94.8	GLIS3	flanking 5′UTR	3.8Kb
**Non-smoker**														
rs17053186	3	53575177	T	0.21	1.64	3.94	1.6×10^−9^	1.83	9.8×10^−9^	0.65	0.0	CACNA1D	intron	−64390
rs1514846	5	17736871	A	0.26	2.01	3.33	3.4×10^−9^	1.87	4.4×10^−10^	0.18	42.4	RP11-454P21.1		
**Liver**														
rs910609	6	44059634	A	0.23	2.71	5.76	2.9×10^−7^	2.49	5.4×10^−8^	0.05	67.5	C6orf223***	flanking 5′UTR	17kb
rs1790349	11	70819998	C	0.22	1.78	7.26	6.2×10^−11^	2.53	3.6×10^−8^	0.37	0.0	DHCR7	flanking 3′UTR	3.1kb
rs3829251	11	70872207	A	0.22	1.54	6.85	9.5×10^−11^	2.38	3.1×10^−7^	0.22	33.3	NADSYN1	intron	−496
rs10898193	11	70874731	T	0.22	1.54	6.82	1.1×10^−10^	2.38	3.2×10^−7^	0.22	34.9	NADSYN1	intron	−389
rs11234042	11	70916734	A	0.22	1.54	6.77	1.4×10^−10^	2.38	3.4×10^−7^	0.21	35.2	KRTAP5-7	3′UTR	242

* The risk is calculated to the risk allele

** Heterogeneity for data from the 3 centers

*** located 11kb 3′ of antisense RNA RP5-112OP11.1

The results on the SNPs from Table 1 and their linked SNPs (r^2^≥0.8) are shown in Supplementary Table S1 based on CADD and HaploReg data. The CADD score was high (18.3, over 10 is considered damaging) for rs9347983 on chromosome 6. The locus was also highly conserved (PhaseCons score 1.0) and was predicted to change binding motifs for 4 transcription factors. rs2660852 flanking 5′UTR of LTA4H was in high LD with rs2540471 located at the binding site of USF1 and NFYB, and changed the motifs of transcription factors Maf, NF-E2 and Nr2f2. The SNP, rs4771282 on chromosome 13 was linked to two conserved SNPs with CADD score over 10. The regulatory features of most other SNPs in Table 1 were changes in transcription factor binding sites.

A regional association plot and functional annotation based on the ENCODE of the smoking-related SNPs from Table 2 provided limited clues to the possible mode of action and no data are shown. However, Regulome and HaploReg data showed that rs10974489 5′ to GLIS3 had a moderate CADD score of 7.6 and it changed a motif for LBP-1 (Supplementary Table S2). rs17053186 mapped among histone marks in fetal lung tissue and changed motifs for Foxp3 and Maf. rs1514846 on chromosome 5 was linked to 4 SNPs with CADD scores of over 10, it mapped among histone marks in A549 lung carcinoma cells and changed binding motifs for SP2 and Znf143.

**Figure 3:**
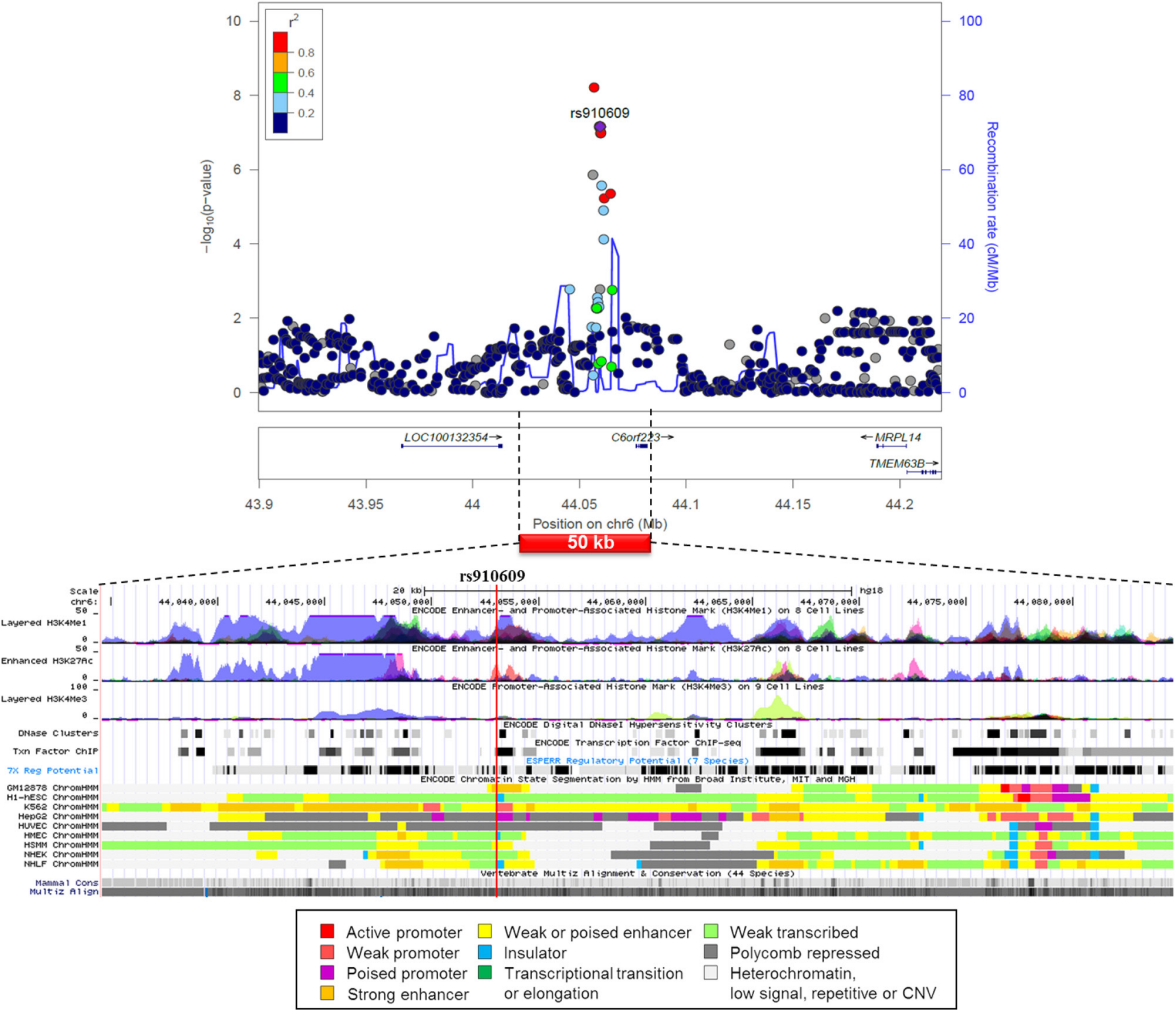
Regional association plot of SNP rs910609 from CUP patients with liver metastasis and controls with functional annotation based on the ENCODE data

A regional association plot and functional annotation based on the ENCODE of rs910609 on the liver-specific association on chromosome 6 showed that the SNP was located in a poised promoter in the hepatocellular carcinoma (HepG2) cells and was mapped among enhancer and promoter histone marks in many cell lines (Figure 3). According to the Regulome and HaploReg data on rs910609, histone marks for chromatin remodeling are found for the liver, the SNP binds P300 and TCF4 and changes motif for AP-2rep (Supplementary Table S2).

**Figure 4:**
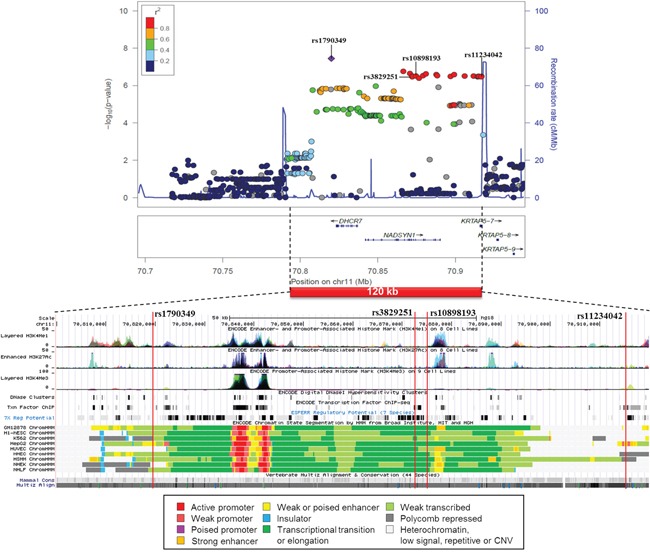
Regional association plot of the 4 SNP cluster on chromosome 11 from CUP patients with liver metastases and controls with functional annotation based on the ENCODE data

A similar association plot and functional annotation is shown for the 4 SNP cluster on chromosome 11 from CUP patients with liver metastases (Figure 4). The sequence between rs1790349 and rs11234042 spans less than 100 kb and covers all the 4 SNPs and numerous other SNPs in high LD. The region is actively transcribed and it contains active promoters and histone activation sites in many cell types, including adult liver and HepG2 cell line. According to the additional Regulome and HaploReg data rs1790349 is located among histone enhancer marks in adult liver and HepG2 cells and changes binding motives of several transcription factors (Supplementary Table S2). This and the other 3 SNPs are eQTLs in many tissues and each change transcription factor binding motives. rs11234042 is located in the 3′UTR and it is only 633 bp from and in high LD with a coding SNP in the KRTAP5-7 gene with a CADD value of 9.9 and a high conservation score of 1.0.

## DISCUSSION

CUP has been considered a heterogeneous phenotype because of its variable clinical presentation and it is speculative to ad hoc propose genetic pathways that may be related to germline risk of CUP. Yet, the association of CUP between family members and also its familial association with many primary cancers suggest that germline genetic factors contribute to this phenotype [13]. Although CUP is not a rare disease - its incidence is higher in Sweden than that of pancreatic cancer - to our knowledge the present patient series is the largest uniform collection of patients. Yet, a total of 578 patients is a not an overwhelming number for a GWAS because, alike other cancers, small relative risks are to be expected [19]. We had no separate verification populations available and thus we made sure that the data from each center was consistent with the findings.

In the association analysis of all CUP, 6 unlinked loci reached a p-value of 10^−6^ -10^−7^ but none reached the generally considered genome-wide significance level of 5×10^−8^, which may reflect genetic heterogeneity of CUP. Typical of the cancer-related GWASs most identified SNPs resided outside coding regions within introns or flanking coding/transcribed regions. However, as a reminder that some 75% of the human genome may be transcribed, 3 SNPs were adjacent to transcribed regions of RNA genes [20]. RP11-300M24.1 and RP11-120E13.1 (chromosomes 6 and 13) are long intergenic non-coding RNAs (lincRNA), which are expressed in many cancer cell lines (Expression Atlas, website: http://www.ebi.ac.uk/gxa/home). Y RNA (chromosome 7) is a novel miscellaneous other RNA (misc RNA) for which functional data are sparse. The proximal protein coding genes included LTA4H (leukotriene A4 hydrolase) on chromosome 12, TIAM1 (T-cell lymphoma invasion and metastases) and KCNJ6 (potassium channel, inwardly rectifying subfamily J, member 6) both on chromosome 21. For the SNPs related to LTA4H and TIAM1 a reasonable amount of functional data suggested important regulatory functions.

LTA4H is an epoxide hydrolase that catalyzes the final step in biosynthesis of the proinflammatory leukotriene B4 which is a strong chemotactic factor for mast cells and neutrophils and has been implicated in the pathogenesis of several chronic inflammatory diseases and of cancer through increasing transcription of oncogenes and interfering with apoptosis [21]. It has been shown that inflammatory markers are elevated in CUP patients [22]. Arachidonic acid is the parent compound for the synthesis of leukotriene B4 as well as of prostaglandins which are metabolized by cyclo-oxygenase (COX). This enzyme is the target of aspirin cancer prevention and it is also known to reduce the risk of CUP [23]. These data are consistent with the notion that inflammation is one of the driving forces in CUP. Protein Tiam1 modulates the activity of RHO-like proteins and it is a regulator of Rac1 mediated signaling pathways connecting extracellular signals to many types of intracellular processes, including membrane trafficking, cell migration, adhesion and invasion, and thus relating to cell growth, survival, metastasis and carcinogenesis [24]. TIAM1 is overexpressed in many tumors, including melanoma, and breast, colon, prostate and renal cancers [25]. KCNJ6 activity may be related to obesity and diabetes [26].

Among analysis of CUP subtypes the genome-wide significance was reached among smokers, non-smokers and patients with liver metastases. The GLIS3 gene that was detected among smokers is a transcription factor regulating the development of liver, kidney and pancreatic beta cells; it is associated with diabetes and also with liver cancer [27].

In patients with liver metastases SNP rs910609 on chromosome 6 flanked 5′UTR of C6orf223, a protein coding gene with unknown function but with a polymorphic variant associated with macular degeneration [28]. The <100 kb cluster of linked SNPs in the chromosome 11 encompassed 3 genes, DHCR7, NADSYN1 and KRTAP5-7. DHCR7 encodes an enzyme which catalyzes the conversion of 7-hydroxycholesterol to cholesterol. Cholesterol promotes cancer signaling, in part, due to the assembly of cholesterol-rich membrane microdomains (lipid rafts) and due to the dependency of rapidly dividing cancer cells on cholesterol and other lipids [29]. Liver is the main site of cholesterol metabolism which may explain why CUP metastases were found in this organ. Sterol metabolic pathways have been promising anticancer targets through examples such as statins and farnesyl transferase inhibitors. NAD (nicotinamide adenine dinucleotide) synthetase catalyzes the final step in the biosynthesis of NAD from nicotinic acid adenine dinucleotide. NAD has a multitude of functions, such as being a coenzyme in metabolic redox reactions, a precursor for several cell signaling molecules, and a substrate for protein posttranslational modifications. SNPs in NADSYN1 regulate serum vitamin D levels but are not related to esophageal or colon cancers or melanoma [30]. Although keratins and keratin associated proteins have numerous links with cancer the data exactly on KRTAP5-7 cannot be found. Notable in this analysis, a linked coding variant was found in KRTAP5-7 and this was predicted to be deleterious. All 4 SNPs had a fair amount of regulatory data, importantly also covering the liver, which all provide supporting evidence on the active role of this locus.

In summary, this first GWAS on CUP showed suggestive associations at the level of 10^−7^ in the flanking 5′UTR of LTA4H, a gene coding for an enzyme catalyzing the production of proinflammatory leukotriene B4. This is an interesting candidate involved in chronic inflammatory processes and cancer. Another gene, TIAM1 (T-cell lymphoma invasion and metastases), is not only involved in lymphoma but in many cancers where Tiam1 is proposed as a prognostic marker for breast, colon and hepatocellular cancer progression and metastasis [24]. Thus it is also an attractive candidate for CUP carcinogenicity. The associations with RNA genes remain to be explored once more functional information on these genes become available. CUP with metastases in the liver showed genome-wide associations for a chromosome 11 region clustering genes DHCR7, NADSYN1 and KRTAP5-7, the first two ones encoding key enzymes in cholesterol and NAD synthesis, and the last one encoding a keratin associated protein. All the three genes would have potential roles in carcinogenesis. These data provide a preliminary notion, in lacking direct functional evidence, for a germline architecture of CUP involving proinflammatory and metastatic gene variants in association with lipid metabolic disturbance.

## PATIENTS AND METHODS

The Swedish patients were recruited from Swedish prospective biobanks (Umea and Malmö). The Umea Medical Biobank included 203 CUP patients with a median diagnostic age of 66 years. The control population was 1055 healthy persons which served as GWAS controls in the Umea Fracture and Osteoporosis (UFO) study; the mean age was 56 years at the baseline [31]. The Malmö Diet and Cancer Study and the Malmö Prevention Study allowed a joint identification of 270 CUP patients with mean diagnostic ages of 71 years. The control population was drawn from these local biobanks, including 5368 cancer-free persons. Cancers were identified up to year 2012 through the regional Oncology Centers that collect data for the Swedish Cancer Registry. The German patients were obtained from the Heidelberg clinic (52 patients with mean diagnostic age 61 years) and from a German CUP trial (53 patients with mean diagnostic age 56 years). Diagnostic years ranged from 2011 through 2013. Controls were 2205 healthy persons of German origin with a mean age of 68 years [32]. On all CUP populations data on sex, age and smoking habits (except the German trial patients) were available. Clinical data included histology and metastatic locations, classified as ‘unspecified CUP’ including spread to multiple organs, ‘liver CUP’ with liver metastasis, ‘lung CUP’ with lung involvement (including thorax and lymph nodes and organs, brain and bone, for which lung cancer is usually given as the cause of death in CUP patients [14, 33]), ‘abdominal CUP’ with abdominal metastases (including ovary) and ‘other CUP’ with other metastatic locations (any other specified site).

All samples were genotyped using Illumina Human Omni1-Quad BeadChips or OmniExpress-12 v1.0 arrays. As to the control metrics, samples were excluded if <95% of SNPs were successfully genotyped or if identity-by-state probabilities for pairs of samples were >0.20. To identify individuals of divergent ancestry we conducted a principal component analysis using smartPCA from the EIGENSTRAT package and a pruned SNP set. SNPs having a minor allele frequency <5% or a call rate <95% were excluded. Consistency of the minor allele frequency (MAF) in the control population was checked to be consistent with the the 1000 Genomes EUR population. SNPs showing departure from Hardy-Weinberg equilibrium in controls at P < 10^−5^ were also excluded. The analyses were conducted using PLINK (v1.07) and EIGENSTRAT software. The possibility of differential genotyping of CUP patients and controls were evaluated using quantile-quantile (Q-Q) plots of test statistics. Odds ratios (ORs) and associated 95% confidence intervals (CIs) were calculated by unconditional logistic regression. Cochran's Q statistic was calculated to test for heterogeneity between data from the three centers and the I^2^ statistic was used to quantify the proportion of the total variation due to heterogeneity; I^2^ values ≥75% are considered a large heterogeneity. For untyped SNPs imputation was carried out to the 1000 Genomes data. However, because we had no possibility to validate imputed SNPs, these were considered only when the imputed SNPs were in linkage disequilibrium with genotyped SNPs.

Regional association plots and the epigenetic profiles of the best associated regions were used to check the chromatin state segmentation profiles (ChromHMM) in 9 cell lines, including lymphoblastoid cells (GM12878) and human liver cancer cell line HepG2, generated by the ENCODE project and available at the UCSC Genome Browser [34]. The possible functional roles of the SNPs were assessed by the ENCODE-based tool HaploReg v4.1 (www.broadinstitute.org/mammals/haploreg). To evaluate the regulatory nature and the possible functional effects of SNPs and their associated SNPs with r^2^ ≥ 0.8, computational predictions were performed using HaploReg v4.1 [35], CADD (combined annotation dependent depletion) [36], Regulome DB [37] and UCSC genome browser [34]. Variants with CADD scores greater than 10 are considered to be deleterious. Variants were visualized in the human genome using the Locuszoom [38] and the UCSC genome browser [34].

The study was approved by the ethics committees at Umea University and Heidelberg University.

## SUPPLEMENTARY FIGURES AND TABLES






