# Supplementary material for: Germline genetics of cancer of unknown primary (CUP) and its specific subtypes
Source: Oncotarget. 2016 Mar 3;7(16):22140–9. doi: 10.18632/oncotarget.7903 (PMC5008350; doi:10.18632/oncotarget.7903)
Supplement: Supplementary file 1 [file oncotarget-07-22140-s001.pdf]

SUPPLEMENTARY TABLES

Supplementary Table S1: Summary of functional and regulatory annotation of most significant SNPs in the case-control study of all CUP patients (from Table 1, herein marked in bold letters) and SNPs that are associated by LD (r^2) ≥ 0.80 using HaploReg V4.1 and CADD V1.3

See Supplementary File 1

Supplementary Table S2: Summary of functional and regulatory annotation of most significant SNPs in analysis of subgroups of CUP patients (from Table 2, herein marked in bold letters) and SNPs that are associated by LD (r^2) ≥ 0.80 using HaploReg V4.1 and CADD V1.3

See Supplementary File 2
